# Modulation of RECK levels in *Xenopus* A6 cells: effects on MT1-MMP, MMP-2 and pERK levels

**DOI:** 10.1186/s40709-019-0108-8

**Published:** 2019-11-27

**Authors:** Jessica A. Willson, Bradley S. Bork, Carlie A. Muir, Sashko Damjanovski

**Affiliations:** Department of Biology, University of Western Ontario, London, ON N6A 5B7 Canada

**Keywords:** A6 cells, RECK, MMP14, MMP2, Zymography

## Abstract

**Background:**

MT1-MMP is a cell-surface enzyme whose regulation of pro-MMP-2 and ERK activation position it as a key facilitator of ECM remodelling and cell migration. These processes are modulated by endogenous MMP inhibitors, such as RECK, a GPI-anchored protein which has been shown to inhibit both MT1-MMP and MMP-2 activity. Our previous studies have revealed a link between MT1-MMP levels, and pro-MMP-2 and ERK activation in mammalian cells, as well as MT1-MMP and RECK co-localization in *Xenopus* embryos. We here investigated how modulation of RECK would impact MT1-MMP and MMP-2 levels, as well as ERK signalling in *Xenopus* A6 cells.

**Results:**

We used a Morpholino approach to knockdown RECK, plasmid transfection to overexpress RECK, and PI-PLC treatment to shed RECK from the cell surface of *Xenopus* A6 cells. RECK reduction did not alter pERK or MT1-MMP levels, nor MMP-2 activity as measured by zymography; thus RECK-knockdown cells maintained the ability to remodel the ECM. RECK overexpression and PI-PLC treatment both increased ECM remodelling potential through increased MT1-MMP protein and relative MMP-2 activation levels.

**Conclusions:**

RECK changes that reduce the ability of the cell to remodel the ECM (overexpression and cell surface shedding) are compensated for by increases in MT1-MMP, and MMP-2 levels as seen by zymography.

## Background

Remodelling of the extracellular matrix (ECM) primarily occurs through the combined action of matrix metalloproteinases (MMPs) and their endogenous inhibitors, tissue inhibitors of metalloproteinases (TIMPs), and reversion-inducing cysteine-rich protein with Kazal motifs (RECK) [1]. The RECK gene encodes a glycosylphosphatidylinositol (GPI)-anchored protein with three protease inhibitor-like domains called Kazal motifs. RECK was initially classified as a tumour suppressor protein due to its ability to suppress metastasis when ectopically expressed in transformed cells [2]. In vitro studies have shown that RECK can inhibit MMP-2 and -9, secreted gelatinases associated with important developmental events and diseases, as well as MT1-MMP, a multi-functional cell surface MMP [2–4]. Many studies have investigated the embryonic role of MMPs. Perhaps due to redundant functions between family members, mice knockouts of each MMP, apart from MT1-MMP, and all TIMPs are viable [5]. This positions MT1-MMP as a uniquely important molecule, and therefore its regulation by other agents, such as RECK, is of interest. Likewise, studies have implicated the importance of RECK during development, as RECK knockout is embryonic lethal. In both mice and fish, loss or reduction of RECK causes defects in angiogenesis and neurogenesis, in part due to aberrant regulation of MMPs [4, 6–8]. In addition, RECK has been shown to play a role in cell migration signalling pathways independent of its MMP-inhibitory role [9].

Our previous studies using epithelial breast cancer cells have revealed a complex pattern of expression between MT1-MMP levels and ERK activation into pERK, as well as pro-MMP-2 activation into MMP-2. Mammalian MCF-7 cells stably expressing extremely high levels of MT1-MMP demonstrated low ERK activation and high pro-MMP-2 activation. Conversely, cells with low levels of MT1-MMP demonstrated high ERK activation and low pro-MMP-2 activation [10]. Treatment with the broad spectrum MMP inhibitor BB94 increased MT1-MMP expression, but decreased MMP-2 activation and did not alter pERK protein levels [11]. In mammalian MDA-MB-231 cells, inhibition of pro-MT1-MMP activation elevated global MT1-MMP and pERK levels, but decreased MMP-2 levels [12]. These mammalian cell studies demonstrated that when MT1-MMP levels are altered, pERK and MMP-2 activation levels alter in different ways depending on treatment. This suggests a mechanism that may involve more molecules than just MT1-MMP in the activation of ERK and pro-MMP-2, with RECK possibly playing an important role. We have previously cloned and characterized the RECK gene and studied its expression during *Xenopus laevis* development, showing that RECK is present at stages where ECM remodeling events are associated with neural function [13]. Our recent in vivo examination of RECK, MT1-MMP, and TIMP-2 show that these proteins colocalize in the dorsal axis of 48-h *Xenopus* tailbud stage embryos, particularly in the neural tube [14]. Several other studies have also described interactions between RECK and MT1-MMP proteins in vivo and in vitro [15–17], with RECK being shown to complex with MT1-MMP at the cell surface to both attenuate its proteolytic activity and modulate its endocytosis from the cell surface [18].

To corroborate our in vitro mammalian work that link MT1-MMP and pERK levels, as well as build on our *Xenopus* in vivo localization of MT1-MMP and RECK, we here used an in vitro examination of *Xenopus* A6 epithelial cells to confirm the importance of RECK as it relates to MT1-MMP, pERK, and MMP-2 protein levels, across poikilotherm and ectotherm species, and between in vivo and in vitro models. In this study, we used a Morpholino approach to knock down RECK levels, plasmid transfection to overexpress RECK, and PI-PLC treatment to shed RECK from the surface of A6 cells. RECK reduction did not alter MT1-MMP protein levels, ERK activation, or MMP-2 activity levels. RECK overexpression and PI-PLC treatment both resulted in increased MT1-MMP protein levels and MMP-2 activity levels. Only RECK overexpression decreased pERK protein levels in A6 cells. From these results, it is suggested that optimal levels of RECK present on the cell surface are important for modulating MT1-MMP protein levels and MMP-2 activation.

## Methods

### Morpholino design

The design and synthesis of Morpholinos (MO) were performed by Gene Tools (Philomath, USA). A translation-blocking MO (antisense: CATCACATCCCCACTCCTTCTCTTC) was engineered to target *X. laevis* RECK (GenBank, AIZ00509.1). Standard scrambled MOs (antisense: CCTCTTACCTCAGTTACAATTTATA) and a carboxyfluoresceinated-labelled MO targeted to the *X. laevis* β-catenin gene (antisense: TTTCAACCGTTTCCAAAGAACCAGG) were also purchased from Gene Tools as controls.

### Cell culture conditions, Endo-Porter treatments, transfections, and PI-PLC treatments

*Xenopus laevis* A6 cells (ATCC^®^CCL-102™) were maintained at 24 °C in L-15 (Leibovitz) medium (Wisent Inc., Saint-Jean-Baptiste, Canada) containing 10% fetal bovine serum (FBS) and 1% penicillin (100 U mL^−1^)/streptomycin (100 µg mL^−1^) [19].

For RECK knockdown, cells were seeded on 35 mm dishes at a density of 1 × 10^6^ cells. After 24 h, spent culture medium was replaced with medium containing Endo-Porter reagent and MO oligos (Gene Tools, Philomath, USA) according to manufacturer’s instructions. Forty-eight hours following treatment, cell lysates were collected. MOs were used in dosages of 1, 10, or 20 µM. Following confirmation of RECK protein decrease, 20 µM MO treatment was used in subsequent experiments.

For RECK overexpression, cells were seeded as above. Twenty-four hours later, cells were transfected with HA-tagged RECK in pcDNA3.1 using Lipofectamine 2000 (Thermo Fisher, Mississauga, Canada) according to manufacturer’s instructions. Another 24 h following transfection, cell lysates were collected. The generation of the full-length RECK cDNA construct is described in [13], with the HA tag being inserted just following the N-terminal signal sequence such that it would not be removed during secretion, nor would it interfere with GPI anchor formation at the C-terminal end.

Phosphatidylinositol-specific phospholipase C (PI-PLC) is an enzyme from *Bacillus cereus* that cleaves GPI-linked proteins, such as RECK, from the plasma membrane. Twenty-four hours following transfection, RECK-transfected and mock-transfected cells were treated with 100 U mL^−1^ of PI-PLC (Thermo Fisher, Mississauga, Canada) in serum-free L-15 media according to manufacturer’s instructions. Twenty-four hours later, cell lysates were collected.

### Quantitative real-time PCR

To investigate changes in transcript levels, real-time qPCR was performed. Cells were seeded and treated as described above. Following treatment, RNA was extracted from cells using an RNeasy Mini Kit (Qiagen, Montreal, Canada) according to manufacturer’s instructions. cDNA was synthesized from 1 µg of RNA using qScript™ cDNA SuperMix (QuantaBio/VWR, Mississauga, Canada) according to manufacturer’s instructions. qPCR was carried out using SYBR Green SuperMix (Applied Biosystems/Thermo, Mississauga, Canada) according to manufacturer’s instructions. For quantification, the target genes (MMP-2, MMP-9, MT1-MMP, and TIMP-2) were normalized to the internal standard of EF-1α. Fold change was calculated according to the ∆∆CT method [20]. Primers used (5΄–3΄): MT1-MMP F: CATGGGCAGCGATGAAGTCT, R: CCAGTATTTGTTCCCCTTGTAGAAGTA; MMP-2 F: CAGGGAATGAGTACTGGGTCTATT, R: ACTCCAGTTAAAGGCAGCATCTAC; MMP-9 F: AATCTCTTCTAGAGACTGGGAAGGAG, R:AGCTGATTGACTAAAGTAGCTGGA; TIMP-2 F: GCCCCCGCCCGCCCAGCC, R: CGAGACCCCACACACTGCCGA; EF-1α F: CAGATTGGTGCTGGATATGC, R: ACTGCCTTGATGACTCCTAG.

### Immunoblot analysis

To investigate changes in RECK, MT1-MMP, and pERK protein levels, immunoblotting was performed. Cells were seeded and treated as described above. Following treatment, cells were washed 2× with PBS (pH 7.2) and disrupted using modified RIPA lysis buffer (150 nM NaCl, 1% NP-40, 0.5% sodium deoxycholate, 0.1% SDS, 50 mM TRIS, pH 8.0) supplemented with phosphatase/protease inhibitors (Thermo Scientific, Mississauga, Canada). Collected cell lysates were shaken on ice for 20 min and sonicated 3× for 10 s each. Protein level was quantified using a BCA assay (Thermo Scientific, Mississauga, Canada). Ten µg of protein per sample was mixed with the appropriate amount of loading dye and subjected to SDS-PAGE at 130 V for 3 h. Protein was then transferred to a PVDF membrane (Bio-Rad, Mississauga, Canada) overnight at 4 °C at 12 V. Membranes were blocked with 0.5% bovine serum albumin (BSA) (Fischer Scientific, Mississauga, Canada) in TBST (pH 7.5) for 30 min at room temperature, and subsequently incubated overnight at 4 °C with primary antibody (RECK, 1:200, Cell Signaling Technology, Danvers, USA; HA, 1:500, Santa Cruz, Dallas, USA; MT1-MMP, 1:200, EMD Millipore, Burlington, USA; pERK, 1:16,000, Cell Signaling Technology, Danvers, USA; total ERK1/2, 1:4000, Cell Signaling Technology, Danvers, USA; β-actin, 1:1000, Santa Cruz, Dallas, USA) followed by incubation for 1 h at room temperature with either HRP-labeled goat anti-rabbit or anti-mouse secondary antibodies (Thermo Fisher Scientific, Mississauga, Canada) diluted 1:10,000 in blocking solution. Signal was detected using a SuperSignal Chemiluminescent kit (Thermo Scientific, Mississauga, Canada) as per the manufacturer’s instructions. Blots were visualized using the Bio-Rad ChemiDoc Imaging system and quantified using Bio-Rad QuantityOne 1-D image analysis software (Bio-Rad, Mississauga, Canada). ERK1/2 activation is presented as a ratio between pERK and total ERK1/2 band intensities.

### Gelatin zymography

To measure the activity of secreted MMP-2 and MMP-9 proteins, gelatin zymography was performed. Cells were seeded and treated as described above. Twenty-four hours later, culture media was replaced with serum-free L-15 media. Twenty-four hours later, serum-free media was collected and mixed with 2× SDS-loading dye (0.5 M Tris–HCl pH 6.8, 10% SDS, 2.5% glycerol, and 1% Bromophenol Blue) in a 1:1 ratio, with only 5 μL of sample being loaded into the gel [21]. To aid in the identification of active versus inactive forms of an MMP, the broad spectrum MMP inhibitor BB94 (10 μM) was added to duplicate samples. Media samples were run onan SDS-containing 15% polyacrylamide gel, co-polymerized with 1% gelatin, at 140 V for 6 h. A Triton X-100 (2.5%) incubation was then used to renature MMP-2 and MMP-9 proteins. Gels were placed in developing buffer (pH 7.5, 50 mM Tris, 200 mM NaCl, 5 mM CaCl_2_, and 0.02% Brij-35) at 37 °C with gentle shaking. After 48 h, gels were stained with 0.5% Coomassie blue for 1 h followed by destaining (50% methanol) for 2 h. Gels were visualized and band intensities representative of enzyme activity quantified using Bio-Rad (Mississauga, Canada) ChemiDoc Imaging system and QuantityOne 1-D image analysis software. As protein used for zymography was isolated from the media, the use of an internal (loading) standard was not possible. Accordingly, the ratio of active over total (pro, intermediate, and active) forms was determined for MMP-2. These ratios (active MMP-2/total MMP-2) were then used to calculate the percentage change of MMP-2 activation levels between control and treated cells.

### Statistical analysis

Statistical analysis and graphing were performed using Microsoft Excel. Data is presented as mean ± SD, except for qPCR results in Fig. 7 which are ± SEM. One-way ANOVA followed by unpaired student’s t-tests were used and indicated respectively in each figure legend. The different levels of significance are denoted as follows: ns, *p *> 0.05; *, *p *≤ 0.05; **, *p *≤ 0.01; ***, *p *≤ 0.001;****, *p *≤ 0.0001.

## Results

### RECK reduction in A6 cells did not alter MT1-MMP or pERK protein levels nor MMP-2 activity levels

**Fig. 1:**
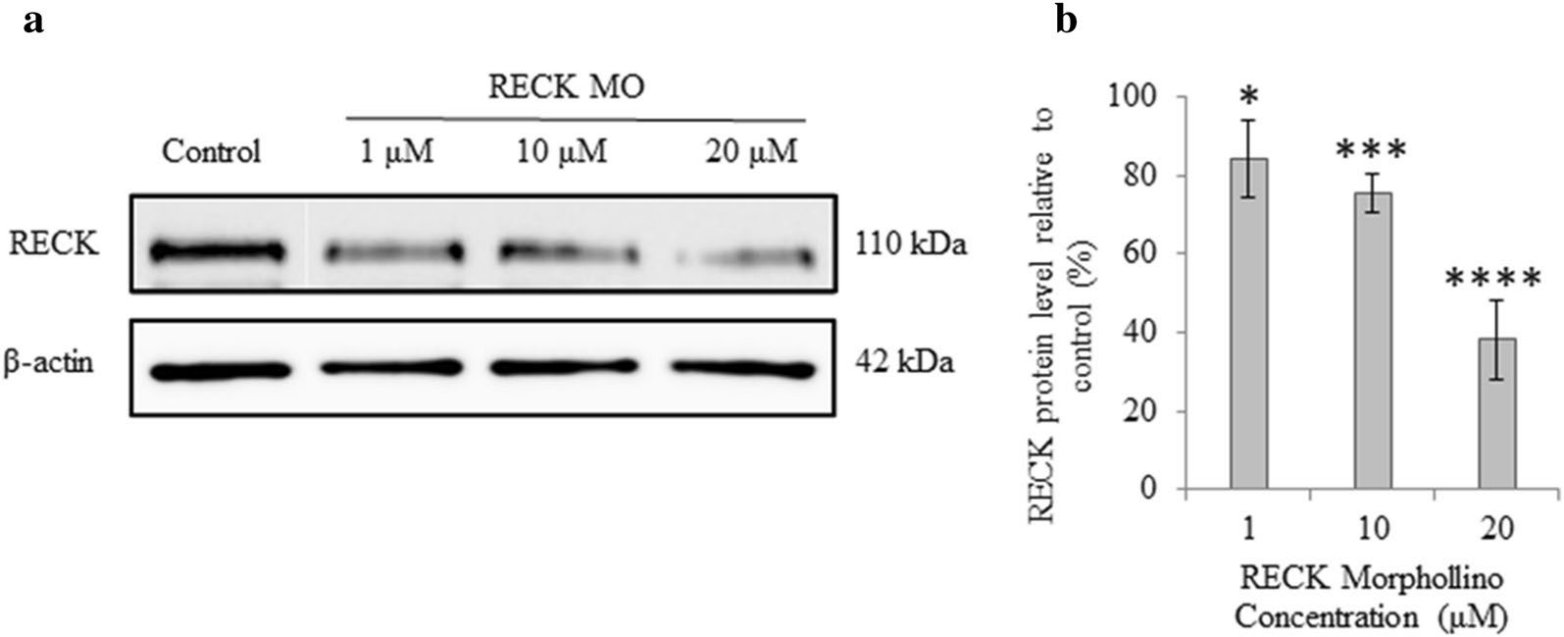
Treatment of A6 cells with RECK MO resulted in decreased RECK protein levels. Immunoblot analysis was used to confirm reduction of RECK protein following treatment of A6 cells with RECK MO using Endo-porter delivery reagent. Protein was extracted from cells 48 h following treatment with increasing concentrations of RECK MO. **a** Knockdown of RECK protein occurred in a dose-dependent manner. β-actin is used as a loading control. **b** Quantification of protein levels in **a** were graphed and data is based on 3 biological replicates (mean ± SD) and normalized to control (set to 100%). Data is analyzed via *t* test; *, *p* ≤ 0.05; ***, *p* ≤ 0.001; ****, *p* ≤ 0.0001

**Fig. 2:**
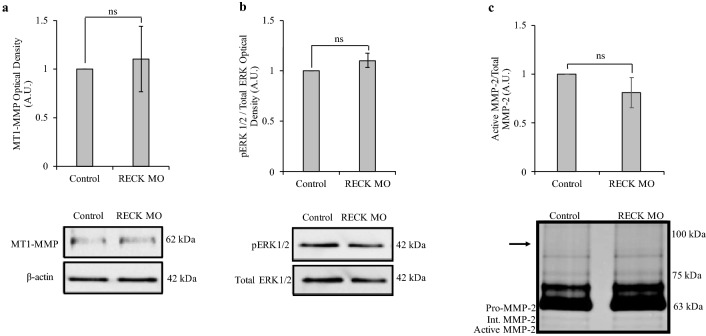
RECK reduction did not alter MT1-MMP or pERK protein levels nor MMP-2 activity levels. **a** Densitometry quantification of MT1-MMP immunoblotted protein normalized to β-actin. MT1-MMP protein levels did not significantly change in RECK knockdown cells compared to control (set to 1). β-actin is used as a loading control. **b** Densitometry quantification of pERK normalized to total ERK levels. pERK protein levels did not significantly change in RECK reduced cells compared to control (set to 1). **c** Gelatin zymography was used to measure protein activity of secreted pro-, intermediate, and active MMP-2 following RECK knockdown in A6 cells. Data is presented as the ratio between active and total (pro-, intermediate, and active) MMP-2 levels between control and RECK reduced cells. Active/total MMP-2 levels did not significantly change in the media of RECK reduced cells compared to control (set to 1). Secreted MMP-9 protein could not be detected at the expected size of 92 kDa (shown by the arrow). Graphed data is based on 3 biological replicates (mean ± SD). Data is analyzed via t-test; ns, *p* > 0.05

To confirm that the RECK MO treatment decreased RECK protein level in *Xenopus* A6 cells RECK protein was quantified following treatment with different concentrations of RECK MO. Immunoblotting demonstrated that RECK protein is endogenously expressed in untreated A6 cells. A dose-dependent decrease in cellular RECK protein level was then seen following treatment of increasing concentrations of RECK MO (Fig. 1a). The decrease in RECK was significant when immunological signals were quantified, with the 20 µM treatment reducing RECK levels by 60% (Fig. 1b). RECK level did not change following treatment of scrambled MO or β-catenin MO [unpublished observations]. Having confirmed the efficacy of the RECK MO, we examined the levels of MMPs and their activity in A6 cells following RECK knockdown. RECK reduction did not alter MT1-MMP nor pERK protein levels (Fig. 2a, b) as seen by immunoblot analysis. However, this 60% reduction in RECK was large enough to cause a cellular effect, as there was a change in transcription levels (see also Fig. 7a).

Zymography was then used to quantify the active and inactive secreted forms of MMP-2 and MMP-9 after RECK reduction. Bands of gelatin degradation representative of MMP activity of specific molecular mass on zymograms were quantified. The addition of the broad spectrum MMP-inhibitor BB94 was used to confirm the identity of active versus pro-MMP forms [unpublished observations]. The amount of the active form of an MMP was compared to the total amount of MMP (pro, intermediate, and active forms). The active/total MMP ratio was set to 1 in control cells. The relative level of MMP-2 activity did not change (there was the same ratio of active to total MMP-2) after RECK reduction (Fig. 2c). MMP-9 activity could not be detected in the media (Fig. 2c) at the previously described molecular mass [10, 22, 23].

### RECK overexpression in A6 cells caused an increase in MT1-MMP protein levels and relative active MMP-2 levels, and a decrease in pERK protein levels

**Fig. 3:**
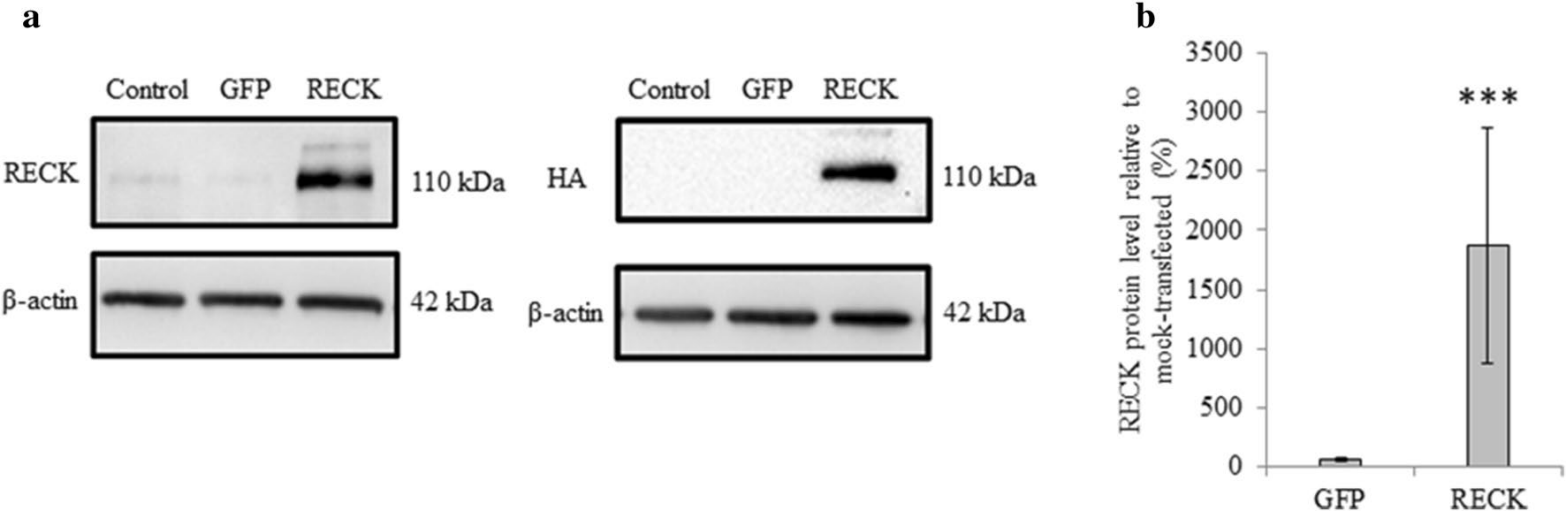
Transfection of full-length HA-tagged RECK constructs in A6 cells resulted in increased RECK levels. **a** Immunoblot analysis was used to confirm overexpression of RECK protein following transfection of RECK cDNA constructs. Mock-transfected and GFP-transfected A6 cells were used as controls. Protein was extracted from cells 24 h following transfection. β-actin is used as a loading control. Left panel demonstrates increased levels of RECK protein compared to control using a RECK antibody, while right panel confirms the presence of HA-tagged proteins only in RECK-transfected A6 cells using an HA antibody. **b** Graphed data is based on 3 biological replicates (mean ± SD) and normalized to control (set to 100%). Data is analyzed via t-test; ***, *p* ≤ 0.001

After observing no change in MT1-MMP, pERK, and MMP-2 activation following RECK reduction, we proceeded with overexpression of RECK in A6 cells. Transfection of an HA-tagged RECK construct was confirmed, as the level of RECK protein in transfected cells significantly increased. This increase in protein was detected and quantified using both a RECK and HA antibody (Fig. 3a, b).

**Fig. 4:**
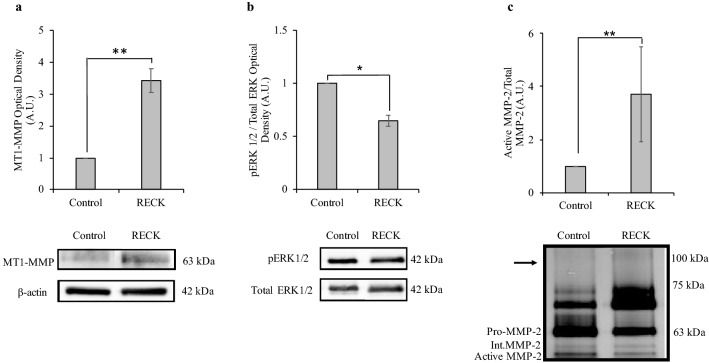
RECK overexpression increased MT1-MMP protein and MMP-2 activity levels and decreased pERK protein levels. **a** Densitometry quantification of immunoblots of MT1-MMP protein normalized to β-actin. MT1-MMP protein levels significantly increased in RECK-overexpressing cells compared to control (set to 1). **b** Densitometry quantification of pERK normalized to total ERK levels. pERK protein levels significantly decreased in RECK-overexpressing cells compared to control (set to 1). **c** Gelatin zymography was used to measure protein levels of secreted pro-, intermediate, and active MMP-2 following RECK overexpression in A6 cells. Data is presented as the ratio between active and total (pro-, intermediate, and active) MMP-2 levels between control and RECK-overexpressing cells. Active/total MMP-2 levels significantly increased in the media of RECK-overexpressing cells compared to control (set to 1). Secreted MMP-9 protein could not be detected at the expected size of 92 kDa (shown by the arrow). Graphed data is based on 3 biological replicates (mean ± SD). Data is analyzed via t-test; *, *p* ≤ 0.05; **, *p* ≤ 0.01

As was done with the knockdown treatment, cellular proteins of interest were quantified following RECK overexpression. Although no change was observed following RECK reduction, RECK overexpression resulted in a significant increase in the level of MT1-MMP protein (Fig. 4a) as well as a significant decrease in the level of pERK protein (Fig. 4b). Following RECK overexpression, zymography of secreted proteins revealed a significant increase in the relative level of active MMP-2 (Fig. 4c). Secreted MMP-9 proteins could not be detected via zymography (Fig. 4c).

### Treatment with PI-PLC reduced cell-surface RECK levels

**Fig. 5:**
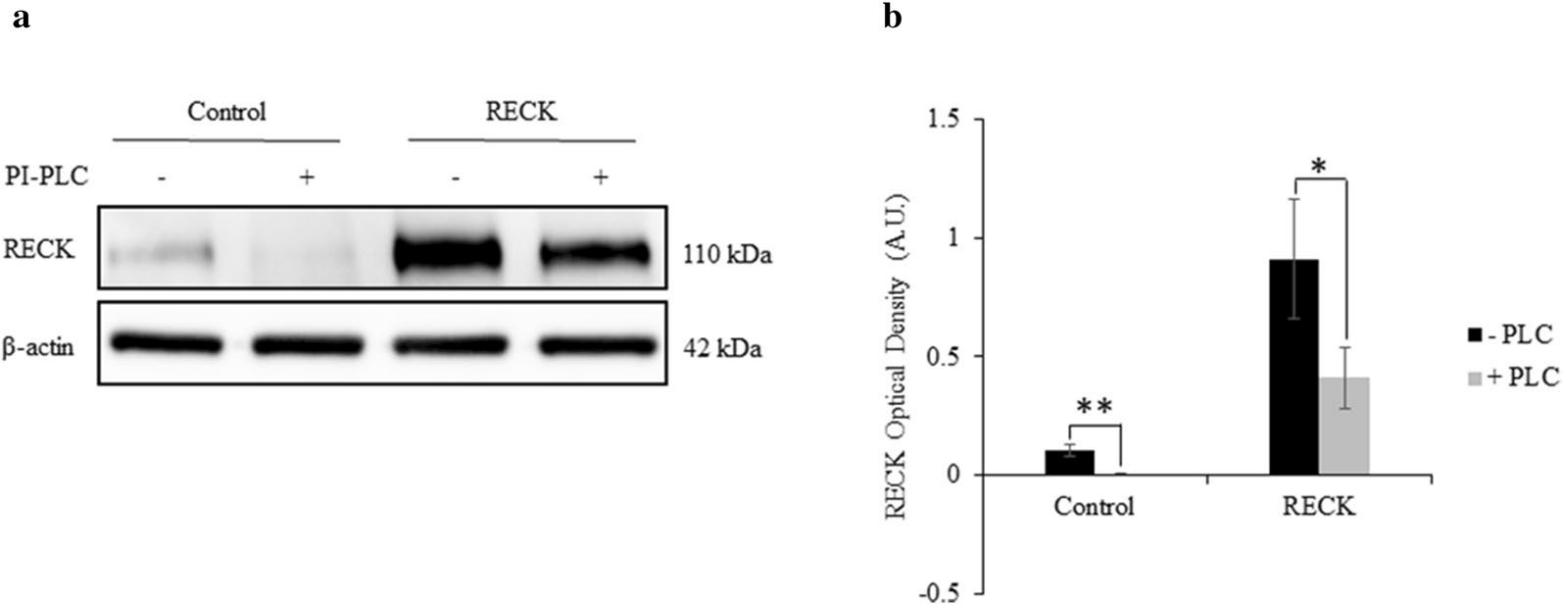
Solubilization of RECK proteins following PI-PLC treatment. **a** Twenty-four hours following transfection of RECK constructs, control and transfected A6 cells were treated with PI-PLC for 24 h and cell lysate collected and analyzed by Western blot. **b** Densitometry quantification demonstrates that the level of RECK proteins bound to the cell is significantly reduced following PI-PLC treatment in both mock-transfected and RECK-transfected cells. β-actin is used as a loading control. Graphed data is based on 3 biological replicates (mean ± SD). Data is analyzed via t-test; *, *p* ≤ 0.05; **, *p* ≤ 0.01

PI-PLC treatment (which releases GPI-linked proteins) was used to shed RECK from the cell surface, and also allowed us to confirm the proper trafficking and localization of overexpressed RECK protein to the cell surface in the A6 cells. As expected, PI-PLC treatment significantly reduced the level of membrane localized RECK, both in control and RECK-overexpressing cells (Fig. 5a, b).

### PI-PLC treatment of A6 cells caused an increase in MT1-MMPand relative active MMP-2 levels

**Fig. 6:**
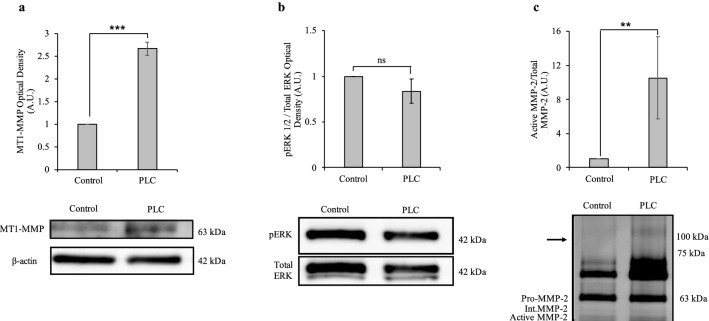
PI-PLC treatment caused an increase in MT1-MMP protein and MMP-2 activity levels. **a** Densitometry quantification of MT1-MMP protein level normalized to β-actin in control A6 cells treated with PI-PLC. MT1-MMP protein levels significantly increased in PI-PLC-treated cells compared to untreated cells (set to 1). **b** Densitometry quantification of pERK normalized to total ERK levels. pERK protein levels did not significantly change following PI-PLC treatment. **c** Gelatin zymography was used to measure protein levels of secreted pro-, intermediate, and active MMP-2 following PI-PLC treatment in A6 cells. Data is presented as the ratio between active and total (pro-, intermediate, and active) MMP-2 levels between untreated cells and PI-PLC treated cells. Active/total MMP-2 levels significantly increased in the media of PI-PLC-treated cells compared to untreated cells (set to 1). Secreted MMP-9 protein could not be detected at the expected size of 92 kDa (shown by the arrow). Graphed data is based on 3 biological replicates (mean ± SD). Data is analyzed via t-test; ns, *p* > 0.05; *, *p* ≤ 0.05; **, *p* ≤ 0.01

**Fig. 7:**
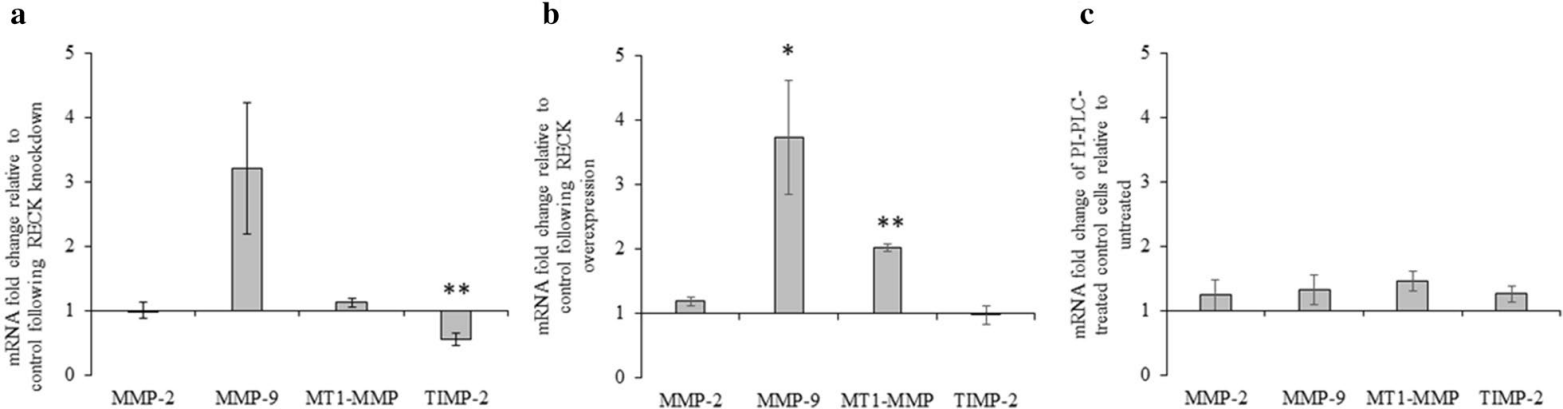
Effect of RECK knockdown, RECK overexpression, and PI-PLC treatment on mRNA levels. The levels of MMP-2, MMP-9, MT1-MMP, and TIMP-2 mRNA were measured following RECK knockdown, overexpression, and PI-PLC treatment in A6 cells using real-time qPCR. **a** Following RECK reduction in cells, MMP-2, -9, and MT1-MMP levels did not change significantly compared to control; however, TIMP-2 levels decreased significantly. **b** Following RECK overexpression, MMP-2 and TIMP-2 mRNA levels did not change significantly, however, MMP-9 and MT1-MMP mRNA levels increased significantly compared to control. **c** Treatment of cells with PI-PLC did not change MMP-2, -9, MT1-MMP, or TIMP-2 mRNA levels. Changes in gene expression were measured relative to EF1α and normalized to control cells (set to 1). Results are based on 3 biological replicates (mean ± SEM; technical replicates, N = 9). Data is analyzed via t-test; *, *p* ≤ 0.05; **, *p* ≤ 0.01

To elucidate the role of RECK at the cell surface in modulating MT1-MMP, pERK, and MMP-2 activation, we shed GPI-anchored proteins, which includes RECK, from the surface using PI-PLC. PI-PLC treatment significantly increased MT1-MMP protein levels (Fig. 6a) but did not alter pERK levels (Fig. 6b). PI-PLC treatment caused an increase in the relative level of active MMP-2 (Fig. 6c). Secreted MMP-9 protein could not be detected (Fig. 6c). Of note—PI-PLC treatment did not alter the transcription of a variety of genes (Fig. 7c), nor did it alter the morphology, growth, or survival of these cells [unpublished observations].

### Alterations in RECK levels or PI-PLC treatment had varying effects on MMP-2, MMP-9, MT1-MMP, and TIMP-2 mRNA levels

Having shown that altered RECK levels had varying effects on protein levels, we sought to better understand the mechanism of these changes by examining changes in RNA levels. Following RECK reduction, qPCR analysis did not reveal a significant change in the level of MMP-2, MMP-9, nor MT1-MMP mRNA. There was, however, a significant decrease in TIMP-2 mRNA levels (Fig. 7a).

Following RECK overexpression, PCR analysis revealed significant increases in MT1-MMP and MMP-9 transcript levels, but no changes in MMP-2 nor TIMP-2 transcript levels (Fig. 7b).

PI-PLC treatment of A6 cells did not alter the transcript levels of MMP-2, MMP-9, MT1-MMP, nor TIMP-2 (Fig. 7c).

Of note, unlike MMP-2, MMP-9 transcript levels in A6 cells were extremely low (only being detectable after 32 PCR cycles) [unpublished observations]. Although MMP-9 transcript levels altered under RECK manipulation (Fig. 7b), we do not believe they elevated enough to be biologically relevant. Indeed, we could not detect MMP-9 proteins secreted by A6 cells via gelatin zymography even when transcript levels increased (Figs. 2c, 4c and 6c).

## Discussion

Adult epithelial cells, unlike their embryonic counterparts, move and invade only under special situations, such as injury or disease. Our in vitro examination of MCF-7 epithelial breast cancer cells, which are poorly invasive, has suggested that MT1-MMP orchestrates this transition towards a more migratory phenotype [9, 10, 24]. While the roles of ECM remodeling proteins have garnered much interest due to their broad developmental and disease implications, it has become clear that there is not a defined relationship between the level of an MMP and the amount of ECM turnover [10]. While not exhibiting overt abilities to remodel the ECM or to become mesenchymal, non-migrating epithelial cells express low levels of the enzymes and signaling molecules needed for these events. Indeed, A6 cells have basal levels of MT1-MMP, MMP-2, RECK and pERK proteins (Figs. 1, 2). It is this low and appropriately localized levels of MT1-MMP and TIMP-2 which can directly regulate enzymatic activity, including the activation of proMMP-2 [11, 25], as well as ERK cell signaling events [10, 11] in mammalian epithelial cells. To address if the maintenance of low levels of MMPs is a common mechanism seen in non-mammalian epithelial cells, here we investigate the impact of altered RECK levels in the context of the ECM remodelers MT1-MMP and MMP-2, as well as ERK in *Xenopus* A6 cells.

The presence of MT1-MMP and active MMP-2 (measured by zymography) demonstrate that A6 cells have at least a basal ability to remodel the ECM. When the endogenous levels of RECK are reduced using MO treatment, no changes are seen in MT1-MMP and pERK protein levels, nor MMP-2 activation (Fig. 2). Despite its ability to act as an MMP inhibitor, reduced levels of RECK were not substantial enough to alter MT1-MMP or MMP-2 levels, nor the basal level of ECM remodelling that may be associated with them. However, this level RECK reduction was biologically relevant as it was enough to decrease TIMP-2 mRNA levels significantly (Fig. 7). Thus, it appears that A6 cells maintain low levels of RECK at the cell surface, levels which when further reduced, do not impact the protein levels of MT1-MMP, MMP-2, or pERK.

As RECK reduction did not alter MT1-MMP, pERK, nor MMP-2 activity levels, we next examined the effect of RECK overexpression in A6 cells. RECK impedes MT1-MMP directly by binding to it and inhibiting its activity [4], however, RECK overexpression resulted in increased MT1-MMP protein and RNA levels (Figs. 4 and 7), and an associated increase in MMP-2 activation (Fig. 4). While A6 cells did not lower their basal ECM remodelling abilities with respect to MT1-MMP and MMP-2 in response to lowered RECK levels, increased RECK did trigger A6 cells to alter MMP levels. This increase in MT1-MMP and MMP-2 levels could be a compensatory response with the cells increasing these MMP levels to maintain a basal ability to remodel the ECM in response to the increased levels of an inhibitor. Further, RECK is associated with increased MT1-MMP endocytosis [18]. Accordingly, increased RECK levels would reduce cell-surface MT1-MMP protein levels, triggering the cell to increase MT1-MMP production. This RECK-mediated increase in MT1-MMP endocytosis rates would also limit the ability of MT1-MMP to activate ERK, thus pERK levels are reduced under RECK overexpression conditions. In the presence of increased levels of RECK, A6 cells attempt to maintain a basal level of ECM remodelling ability by increasing MMP levels.

While A6 cells could tolerate the reduction of RECK as evident in their maintained MT1-MMP and MMP-2 levels, we next sought to examine the necessity for RECK to be membrane anchored. PI-PLC treatment was used to shed RECK and other GPI-linked proteins from the cell surface. Our PI-PLC treatment of A6 cells did not alter gene expression (Fig. 7), nor did it alter the morphology, growth, or survival of these cells [unpublished observations]. By these measures, PI-PLC treatment of A6 cells was not detrimental, while immunoblot analysis did reveal a significant decrease in cell surface RECK levels (Fig. 5).

Like RECK overexpression, PI-PLC treatment of A6 cells elevated both MT1-MMP protein and MMP-2 activation levels (Fig. 6). A possible mechanism that may explain this is that while cell surface RECK may play a role in MT1-MMP endocytosis [18], solubilized forms of RECK cannot. Following PI-PLC treatment and RECK shedding, less MT1-MMP is internalized and recycled, with more remaining at the cell surface. This increased level of MT1-MMP can then also facilitate higher levels of MMP-2 activity. Indeed, the increase in MT1-MMP protein levels despite unaltered MT1-MMP mRNA changes (Fig. 7) can be explained by soluble RECK proteins not being able to recycle cell-surface MT1-MMP, independent of transcriptional changes.

Unlike RECK overexpression which decreased pERK levels possibly due to increased MT1-MMP recycling, PI-PLC treatment, which would reduce MT1-MMP recycling, did not alter pERK protein levels in A6 cells (Fig. 6). These results suggest that cell surface localization of RECK proteins are important in modulating MT1-MMP function.

## Conclusions

*Xenopus* A6 cells contain RECK protein, as well as pERK, MT1-MMP and MMP-2 levels that facilitate ECM remodeling activity as seen by zymography. RECK reduction did not alter MT1-MMP, pERK, nor MMP-2 activation and did not lower the ability of the cell to remodel the ECM. Unlike the reduction in RECK levels, RECK changes (overexpression and cell surface shedding) that would reduce the ability of the cell to remodel the ECM are compensated for by increases in MT1-MMP and MMP-2 levels as the cell tries to maintain a basal ability to remodel the ECM. A6 and other epithelial cells may attempt to maintain basal abilities to remodel the ECM even when challenged by changes in MMP inhibitor levels.

## Data Availability

Please contact the corresponding author (SD) for information on obtaining the reagents utilized in this publication.
